# Notification of changes in taxonomic opinion previously published outside the IJSEM. List of Changes in Taxonomic Opinion no. 41

**DOI:** 10.1099/ijsem.0.006561

**Published:** 2025-01-31

**Authors:** Aharon Oren, Markus Göker

**Affiliations:** 1 The Institute of Life Sciences, The Hebrew University of Jerusalem, The Edmond J. Safra Campus, Jerusalem 9190401, Israel; 2 Leibniz Institute DSMZ – German Collection of Microorganisms and Cell Cultures, Inhoffenstrasse 7B, 38124 Braunschweig, Germany

The International Code of Nomenclature of Prokaryotes (ICNP) [1] deals with the nomenclature of prokaryotes. This may include existing names (the Approved Lists of Bacterial Names) as well as new names and new combinations. In this sense, the Code is also dealing indirectly with taxonomic opinions. However, as with most codes of nomenclature, there are no mechanisms for formally recording taxonomic opinions that do not involve the creation of new names or new combinations. In particular, it would be desirable for taxonomic opinions resulting from the creation of synonyms or emended descriptions to be made widely available to the public. In 2004, the Editorial Board of the *International Journal of Systematic and Evolutionary Microbiology* (*IJSEM*) agreed unanimously that it was desirable to cover such changes in taxonomic opinions (i.e. the creation of synonyms or the emendation of circumscriptions) previously published outside the *IJSEM* and to introduce a List of Changes in Taxonomic Opinion [2]. Scientists wishing to have changes in taxonomic opinion included in future lists should send a PDF file of the relevant publication to the *IJSEM* Editorial Office or to the Lists Editors.

It must be stressed that the date of proposed taxonomic changes is the date of the original publication, not the date of publication of the list. Taxonomic opinions included in the List of Changes in Taxonomic Opinion do not affect the status of a name under the ICNP [2] and can neither be considered as approved nor as disapproved by the International Committee on Systematics of Prokaryotes. The names that are to be used are those that are the ‘correct names’ (in the sense of Principle 6 of the ICNP) in the opinion of the microbiologist, with a given circumscription, position and rank. A particular name, circumscription, position and rank do not have to be adopted in all circumstances. Consequently, the List of Changes in Taxonomic Opinion must be considered as a service to microbiology, and it has no ‘official character’ other than providing a centralized point for registering/indexing such changes in a way that makes them easily accessible to the scientific community.

The List Editors reserve the right not to include in the list proposed emendations that are in our opinion unimportant and do not significantly alter the original description of the taxon. This includes the addition of new members to the taxon, reclassification of members in a different taxon, addition of genome sequence data and other changes that marginally change the taxon circumscription [3]. Conversely, the mere inclusion of an emendation in a List of Changes in Taxonomic Opinion does not indicate that the List Editors regard this emendation as taxonomically relevant.

**Table:** 

Name/authors	Proposed as	Reference
*Acetobacteraceae* (ex Henrici 1939) Gillis and De Ley 1980 emend. Degli Esposti *et al*. 2023, 19∗	emend.	[4]
*Acidilobaceae* Prokofeva *et al*. 2009 emend. St. John and Reysenbach 2024, 12∗	emend.	[5]
*Aedoeadaptatus* Hitch *et al*. 2022 emend. Malhotra *et al*. 2024, 13∗	emend.	[6]
*Amycolatopsis niigatensis* Ding *et al*. 2007 pro synon. *Amycolatopsis echigonensis* Ding *et al*. 2007	synon.	[7]
*Azomonas* Winogradsky 1938 (Approved Lists 1980) emend. Rudra and Gupta 2024, 26∗	emend.	[8]
*Azotobacter* Beijerinck 1901 (Approved Lists 1980) emend. Rudra and Gupta 2024, 26∗	emend.	[8]
*Caldicoprobacter faecalis* (Winter *et al*. 1988) Bouanane-Darenfed *et al*. 2015 pro synon. *Caldicoprobacter oshimai* Yokoyama *et al*. 2010	synon.	[9]
*Caldicoprobacter oshimai* Yokoyama *et al*. 2010 emend. Bouznada *et al*. 2024, 4∗	emend.	[9]
*Chryseomonas* Holmes *et al*. 1986 emend. Rudra and Gupta 2024, 26∗	emend.	[8]
*Corticibacterium* Li *et al*. 2016 emend. Li *et al*. 2024, 12∗	emend.	[10]
*Corticibacterium populi* Li *et al*. 2016 emend. Li *et al*. 2024, 12∗	emend.	[10]
*Curtobacterium albidum* (Komagata and Iizuka 1964) Yamada and Komagata 1972 (Approved Lists 1980) pro synon. *Curtobacterium citreum* (Komagata and Iizuka 1964) Yamada and Komagata 1972 (Approved Lists 1980)	synon.	[11]
*Curtobacterium citreum* (Komagata and Iizuka 1964) Yamada and Komagata 1972 (Approved Lists 1980) emend. Osdaghi *et al*. 2024, 10∗	emend.	[11]
*Halobellus ramosii* Pérez-Davó *et al*. 2015 pro synon. *Halobellus inordinatus* Qiu *et al*. 2013	synon.	[12]
*Haloferax alexandrinum* corrig. Asker and Ohta 2002 pro synon. *Haloferax volcanii* (Mullakhanbhai and Larsen 1975) Torreblanca *et al*. 1986	synon.	[12]
*Haloferax lucentense* corrig. Gutierrez *et al*. 2004 pro synon. *Haloferax volcanii* (Mullakhanbhai and Larsen 1975) Torreblanca *et al*. 1986	synon.	[12]
*Ignisphaera* Niederberger *et al*. 2006 emend. Podosokorskaya *et al*. 2024, 6∗	emend.	[13]
*Mesorhizobium* Jarvis *et al*. 1997 emend. Li *et al*. 2024, 12∗	emend.	[10]
*Mesorhizobium sophorae* De Meyer *et al*. 2016 emend. Li *et al*. 2024, 12∗	emend.	[10]
*Methanospirillum hungatei* corrig. Ferry *et al*. 1974 (Approved Lists 1980) emend. Pradel *et al*. 2024, 21∗	emend.	[14]
*Muricoccus* Kämpfer *et al*. 2003 emend. Liu and Xin 2024, 8∗	emend.	[15]
*Microbispora amethystogenes* Nonomura and Ohara 1960 (Approved Lists 1980) emend. Bouras and Machado 2024, 6∗	emend.	[16]
*Microbispora bryophytorum* Li *et al*. 2015 emend. Bouras and Machado 2024, 7∗	emend.	[16]
*Microbispora fusca* Zhao *et al*. 2020 pro synon. *Microbispora triticiradicis* Han *et al*. 2018†	synon.	[16]
*Microbispora rosea* Nonomura and Ohara 1957 (Approved Lists 1980) emend. Bouras and Machado 2024, 8∗	emend.	[16]
*Microbispora triticiradicis* Han *et al*. 2018 emend. Bouras and Machado 2024, 8∗	emend.	[16]
*Paracraurococcus* Saitoh *et al*. 1998 emend. Kingkaw *et al*. 2024, 8∗‡	emend.	[17]
*Peptoniphilus porci* Wylensek *et al*. 2021 pro synon. *Peptoniphilus faecalis* Ryu *et al*. 2021§	synon.	[6]
*Plesiomonas shigelloides* corrig. (Bader 1954) Habs and Schubert 1962 (Approved Lists 1980) emend. Duman *et al*. 2024, 14∗	emend.	[18]
*Pontivivens* Park *et al*. 2015 emend. Huang *et al*. 2024, 15∗	emend.	[19]
*Pseudaminobacter* Kämpfer *et al*. 1999 emend. Li *et al*. 2024, 12∗	emend.	[10]
*Pseudomaricurvus* Iwaki *et al*. 2014 emend. Montecillo *et al*. 2024, 8∗	emend.	[20]
*Pseudomonas canavaninivorans* Hauth *et al*. 2022 pro synon. *Pseudomonas alvandae* Girard *et al*. 2022¶	synon.	[21]
*Pseudomonas psychrotolerans* Hauser *et al*. 2004 pro synon. *Pseudomonas oryzihabitans* Kodama *et al*. 1985¶	synon.	[21]
*Pseudomonas zarinae* Girard *et al*. 2022 pro synon. *Pseudomonas ogarae* Garrido-Sanz *et al*. 2022¶	synon.	[21]
*Raoultella* Drancourt *et al*. 2001 emend. Brady *et al*. 2024, 8∗	emend.	[22]
*Scandinavium* Marathe *et al*. 2020 emend. Park *et al*. 2024, 8∗	emend.	[23]
*Serpens* Hespell 1977 (Approved Lists 1980) emend. Rudra and Gupta 2024, 26∗	emend.	[8]
*Sporosarcina* Kluyver and van Niel 1936 (Approved Lists 1980) emend. Yang *et al*. 2024, 292#	emend.	[24]
*Stutzerimonas* Lalucat *et al*. 2022 emend. Rudra and Gupta 2024, 27∗	emend.	[8]
*Teichococcus* Kämpfer *et al*. 2003 emend. Liu and Xin 2024, 9∗	emend.	[15]
*Thermanaerothrix* Grégoire *et al*. 2025 emend. Zayulina *et al*. 2024, 10∗	emend.	[25]
*Winogradskyella poriferorum* Lau *et al*. 2005 emend. Fu *et al*. 2024, 9∗	emend.	[26]
*Xanthomonas euvesicatoria* Jones *et al*. 2006 emend. Constantin *et al*. 2016 emend. Harrison *et al*. 2023, 36	emend.	[27]
*Xanthomonas vasicola* Vauterin *et al*. 1995 emend. Studholme *et al*. 2020, 1158	emend.	[28]

∗The journal in which the name was effective published does not have continuous pages.

†The authors gave *Microbispora fusca* NEAU-HEGS1-5 pro synon. *Microbispora triticiradicis* NEAU-HRDPA2-9.

‡The authors attributed the name to Saitoh and Nishimura (1998).

§The authors proposed *Peptoniphilus porci* Wylensek *et al*. 2021 pro synon. *Peptoniphilus faecalis* Ryu *et al*. 2021. However, *P. porci* has priority, so they should have proposed *Peptoniphilus faecalis* Ryu *et al*. 2021 pro synon. *Peptoniphilus porci* Wylensek *et al*. 2021.

¶The authors presented the synonymy proposal as an emendation.

#The authors attributed the name to Yoon *et al*. 2001.

For references to Lists of Changes in Taxonomic Opinion no. 1–40, see *Int J Syst Evol Microbiol* **55** (2005) 7, 1403; **56** (2006) 11, 1463; **57** (2007) 4, 1376; **58** (2008) 5, 1515; **59** (2009) 5, 1559; **60** (2010) 5, 1482; **61** (2011) 4, 1504; **62** (2012) 7, 1448; **63** (2013), 8, 2371, **64** (2014), 8, 2191; **65** (2015), 7, 2028; **66** (2016), 7, 2469; **67** (2017), 7, 2081; **68** (2018), 7, 2137; **69** (2019), 13, 1850; **70** (2020), 9, 4061; **71** (2021), 004596, 004816; **72** (2022), 005164, 005413; **73** (2023), 005696, 005923; **74** (2024), 006145, 006482.

## Abbreviations

ICNP: International Code of Nomenclature of Prokaryotes
*IJSEM*: *International Journal of Systematic and Evolutionary Microbiology*
